# Serum biochemistry panels in African buffalo: Defining reference intervals and assessing variability across season, age and sex

**DOI:** 10.1371/journal.pone.0176830

**Published:** 2017-05-04

**Authors:** Claire E. Couch, Morgan A. Movius, Anna E. Jolles, M. Elena Gorman, Johanna D. Rigas, Brianna R. Beechler

**Affiliations:** 1 Oregon State University, Department of Integrative Biology, Corvallis, Oregon, United States of America; 2 Oregon State University, College of Veterinary Medicine, Corvallis, Oregon, United States of America; 3 Utah State University, School of Veterinary Medicine, Logan, Utah, United States of America; Universidade de Aveiro, PORTUGAL

## Abstract

Serum biochemical parameters can be utilized to evaluate the physiological status of an animal, and relate it to the animal’s health. In order to accurately interpret individual animal biochemical results, species-specific reference intervals (RI) must be established. Reference intervals for biochemical parameters differ between species, and physiological differences including reproductive status, nutritional resource availability, disease status, and age affect parameters within the same species. The objectives of this study were to (1) establish RI for biochemical parameters in managed African buffalo (*Syncerus caffer*), (2) assess the effects of age, sex, pregnancy, and season on serum biochemistry values, and (3) compare serum biochemistry values from a managed herd to a free-ranging buffalo herd and to values previously published for captive (zoo) buffalo. Season profoundly affected all biochemistry parameters, possibly due to changes in nutrition and disease exposure. Age also affected all biochemical parameters except gamma glutamyl transferase and magnesium, consistent with patterns seen in cattle. Sex and reproductive status had no detectable effects on the parameters that were measured. The biochemical profiles of managed buffalo were distinct from those observed in the free-ranging herd and captive buffalo. Biochemical differences between buffalo from captive, managed, and free-ranging populations may be related to nutritional restriction or lack of predation in the context of management or captivity. The reference intervals provided in this study, in addition to the seasonal and age-related patterns observed, provide a foundation for health investigations that may inform management strategies in this ecologically and economically important species.

## Introduction

Accurate methods for monitoring wildlife health are important for wildlife management, and for predicting threats to human and domestic animal health. Serum biochemistry parameters are often used to detect physiological abnormalities in animals that may reflect disease, but typical values for these parameters vary between populations, age classes, and across season. Thus, serum biochemistry values can only be used to measure wildlife health if typical serum biochemistry values for the population of interest are known.

Serum biochemistry parameters can vary significantly depending on individual age, sex, diet, and reproductive status. For instance, neonates and juveniles are known to have different biochemical parameters compared to adults, related to bone growth, an immature immune system, and distinct metabolism of nutrients [1–7]. Additionally, sex-related differences are often attributed to reproductive cycles or behavioral difference between the sexes [1,2]. Findings vary between species and studies [1,7] which may be due to different management systems or environments, or due to methodological discrepancies in sampling and storage procedures [8].

Season affects resource availability and reproductive cycles, leading to changes in nutrition, immunity, and behavior of foraging animals, which can result in significant effects on disease dynamics [9,10]. These seasonal patterns can lead to systemic biochemical fluctuations, such as changes in protein [11] and mineral [12] concentrations. It is important for disease monitoring purposes to be aware of normal seasonal variation in physiology. As shown by Perez et al, the use of statistical models can help to distinguish between the effects of disease and other seasonal effects on physiological parameters [13].

Environmental conditions such as nutrition, predation, and disease patterns may also differ between captive and wild populations, which may be reflected in variations of the serum biochemical parameters. Differences in biochemistry parameters between captive and free-ranging populations have been demonstrated for several wildlife species [14–17]. These differences are likely due to species-specific responses to capture and captivity, ranging from zoos to managed game parks. Additionally, free ranging and captive animals often have dramatically different diets, which can be reflected in serum levels of essential minerals such as calcium and phosphorus [18–19]. Disease is more intensively controlled in captive animals than in free-living populations, and differences in disease management strategies could contribute to altered biochemical parameters [20]. As such, it is crucial to consider the complex influences of environment factors on the physiological status of wildlife when interpreting biochemical parameters for wild animals.

African buffalo (*Syncerus caffer*) are valuable game animals that contribute to the economies of many countries in sub-Saharan Africa, notably through ecotourism, big game hunting, and game meat. On the other hand, they are natural reservoirs for several diseases that are important at the wildlife-livestock interface, including foot-and-mouth disease virus (FMDV), theileriosis, and brucellosis [21]. Although biochemical parameters have not previously been compared between free-ranging and captive buffalo, Beechler et al [22] demonstrated that hematological parameters vary between these two groups. Establishing reference intervals (RI) for buffalo biochemical parameters provides a valuable tool for detecting changes in herd health, which could have important implications for management decisions. For instance, if nutritional parameters such as magnesium or phosphorous are decreased in captive buffalo, it could indicate that captive management methods need to be altered. If enzymes related to muscle damage are elevated, it may indicate capture stress and perhaps the need to seek alternative capture methods. Prior to this work, RI for African buffalo have been available for hematologic parameters [22] but not for biochemical parameters. Limited biochemical ranges have been published by Species360, which synthesizes opportunistically collected samples in zoos worldwide [23], but sample size in this database is too small to calculate RI. The objectives of this study were to (1) establish RI for serum biochemical parameters in managed African buffalo, (2) assess the effects of age, sex, pregnancy and season (early dry through late wet) on serum biochemistry values, and (3) compare the serum biochemistry values between a managed herd, a free-ranging herd, and previously published values for captive (zoo) buffalo.

## Methods

African buffalo included for this study (n = 418) were located within Kruger National Park (KNP), a 19,000 km^2^ preserve located in northeastern South Africa which contains approximately 30,000 free-ranging buffalo [24]. Two separate populations were sampled for this study, and we also reference Species360 values for 20 captive buffalo for comparison [23]. The majority of our analyses were performed on a managed herd contained within a 900 hectare enclosure in the central area of KNP. Approximate size of this herd at any given time was between 50–65 due to births and deaths, and total number of animals sampled throughout the study period from the managed herd was 101. The managed herd was captured and sampled at 2–3 month intervals between February 2014 and August 2015 as part of an ongoing study on foot-and-mouth disease dynamics in buffalo. Four seasons were assigned based on rainfall patterns in KNP [25] with November-January designated early wet season, February-April designated late wet season, May-July designated early dry season and August-October designated late dry season (Table 1). Average rainfall and average temperature in the study area are highest during the early wet season (261 cm, 26 C), and decrease through the late wet (198 cm, 25.2 C) and early dry (26 cm, 18.8 C) before increasing again slightly during the late dry (62 cm, 21.4 C) [26]. A subset of data from this managed herd was used to generate RI, and evaluate the effects of season, age, sex and pregnancy on the biochemical parameters listed in Table 2. A second analysis compared the values obtained from the managed herd to samples obtained from an age-targeted cohort of 317 female free-ranging African buffalo followed for 4 years and captured and sampled every 6 months from 2008–2012 in southern KNP as part of a study on bovine tuberculosis [27]. All buffalo in both herds were marked so that individuals could be followed over time.

**Table 1:** Sample collection details. The number of samples collected from each age group across four seasons in a herd of managed buffalo contained in a 900 hectare enclosure within KNP.

Season	Capture Month & Year	Calves <1 year	Subadults 1–5.5 years	Adults 5.5–15 years	Geriatrics >15 years
Early Dry	June 2014 June 2015	42	26	58	14
Late Dry	August 2014 August 2015 October 2014	49	43	83	20
Early Wet	December 2014	12	13	24	5
Late Wet	February 2014 February 2015	23	27	42	13

**Table 2:** Serum biochemistry parameters. Eleven biochemistry parameters commonly used in diagnostics were measured in this study and used to generate reference intervals.

Parameter	Unit of Measurement	Description	Chemical Methods [34]	Relevant Citations
Albumin, Alb	g/dL	Protein generated by the liver that can change with liver disease, nutrition status, and hydration status	Dye binding with Bromcresol green.	[38, 39]
Globulins, Glob	g/dL	Proteins that function as carriers, enzymes, complement, and antibodies. May change with inflammation and hydration status	Calculated from total protein—albumin	
Total Protein, TP	g/dL	Albumin plus globulins	Protein forms a complex with copper in the presence of hydroxide, from which absorbance is measured.	
Alkaline Phosphatase, ALP	U/L	Enzyme produced in the liver and bone. Changes can result from liver and bone disease.	Measures rate at which ALP hydrolyzes ρ-Nitrophenyl Phosphate	[5, 39]
Aspartate Aminotransferase, AST	U/L	Enzyme produced in the liver and muscle. A change can result from liver and muscle damage.	Measures rate of NAD+ formation due to oxidation of product formed when AST catalyzes reaction of L-aspartate + α-ketoglutarate.	
γ-Glutamyltransferase, GGT	U/L	Enzyme produced primarily in the liver and sensitive to biliary disease	GGT catalyzes the transfer of a glutamyl group from L- γ-glutamyl-3-carboxyl-4-nitroanilide to Gly-gly. Rate of 3-carboxy-4-nitroaniline production is measured.	
Creatine kinase, CK	U/L	Enzyme specific to muscle tissue. An increase signifies muscle damage.	Dephosphorylates Creatine Phosphate. Changes in absorbance due to NADPH production.	[34,39]
Blood Urea Nitrogen, BUN	g/dL	A byproduct of protein metabolism that is filtered by the kidneys	A coupled reaction of Urea+H_2_O. Rate of NAD^+^ formation is directly proportional to amount of Urea in sample.	[5, 39]
Calcium, Ca	mg/dL	Most common mineral in the body, needed for muscle contraction, bone and tooth formation, cardiac function, and blood clotting.	Binds with Arsenazo III to form calcium-dye complex	[5, 39, 40]
Magnesium, Mg	mg/dL	Mineral needed for muscle and nerve function, development of bone and teeth	Mg activates hexokinase to phosphorylate glucose. Mg concentration measured by increase in NADPH absorbance	[39]
Phosphorous, P	mg/dL	Mineral needed for muscle and nerve function, development of bone and teeth	A coupled reaction forms NADH and other products from sucrose and P. Amount of NADH formed is measured.	[5, 39]

### Managed herd sample collection

**Fig 1:**
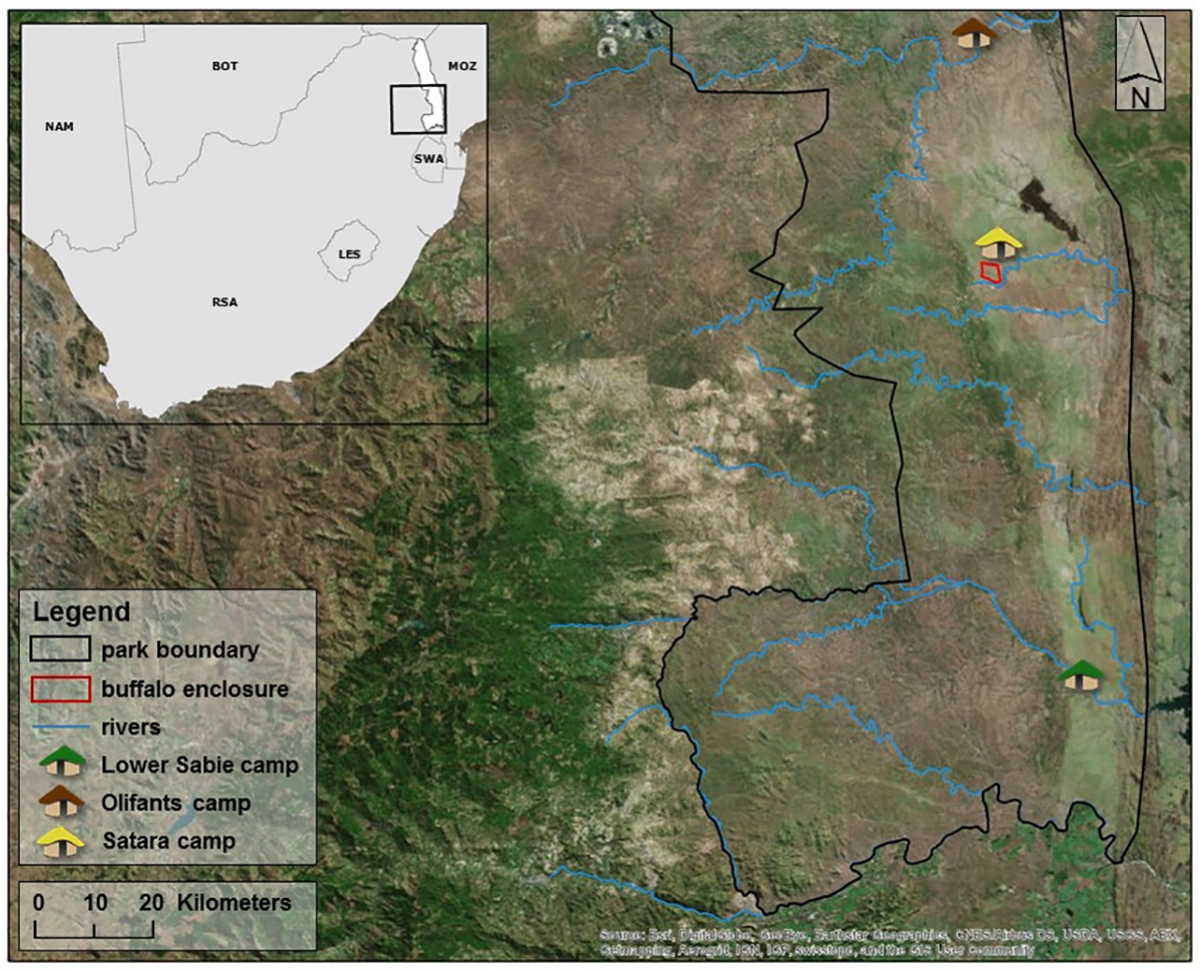
The location of African buffalo used in this study. The enclosure is located in the central portion of KNP near Satara and experiences only 500mm of rain per year, with the majority occurring during the summer months. Total size of the enclosure is 900 hectares, and is enclosed in a double fence to exclude large predators. The buffalo captured as part of the free-ranging herd were initially located in the south-eastern portion of the park centered around Lower Sabie, which experiences similar rainfall to the managed herd. The free-ranging herd was allowed to disperse as normal over the 4 years of the study which resulted in individuals being re-captured throughout the southern portion of the park, as far north as Olifants and spanning the geographic area the managed herd is maintained in [25].

Buffalo for this study were captured in Northern KNP during the early 2000’s and moved to an enclosure near Satara camp (Fig 1). The 900-hectare enclosure includes 50–65 buffalo and other herbivores and small mammalian predators typical of the ecosystem. Large herbivores (e.g. rhinoceros, elephants) and large predators (e.g. lions, leopards) are absent from the enclosure. Water is available to the buffalo at a natural pan (a seasonal pond) and a man-made water reservoir. Buffalo graze and breed as they would in the wild. The current study spans the period from February 2014 to August 2015. Data from a total of 8 captures taken every 2–3 months are included in this analysis. There were a total of 494 samples collected from 101 individual buffalo. Distribution of age categories (see demographic methods for details) by season is shown in Table 1. To sample the buffalo, they were herded into a capture corral, separated into groups of 4–10 animals, and sedated for data collection. Buffalo that could not be herded into the corral were darted individually from a helicopter. Sedation was achieved with 7–10 ug etorphine hydrochloride and 0.04–0.07 mg azaperone per kg body weight for each buffalo. Blood was collected via jugular venipuncture with an 18-gauge needle directly into vacutainer tubes without additives within 5–30 minutes of sedation, and stored on ice for transport back to the laboratory. At the laboratory (approximately 6–8 hours post collection), blood was centrifuged at 5000g for 10 minutes and serum pipetted off the cellular layer into sterile microcentrifuge tubes and stored at -80°C until analysis. All animal work for this study was approved by the institutional animal care and use committee at Oregon State University, ACUP #4478 and at Kruger National Park.

### Free-ranging herd sample collection

Between June 2008 and August 2012, an age-targeted cohort (median age 3.2 years, range 1–15 years) of female African buffalo were captured every 6 months for 4 years in KNP as part of a study on bovine TB [27–31]. Two hundred animals were originally captured from 3 locations spread across the Southern portion of KNP in 2008. Buffalo were re-captured and blood collected every 6 months until 2012. Over the course of the study, 117 animals died and were replaced with similarly aged animals for the duration of the study, resulting in 1750 total samples from 317 individuals. Blood was collected and serum obtained as described for the managed herd. This study was approved by the animal care and use committees at Oregon State (ACUP 3267), University of Montana (AUP 027-05VEDBS-082205), and Kruger National Park.

### Demographic parameter collection

For both studies, age in years was calculated from incisor emergence and tooth wear [32], while sex was determined visually. Pregnancy status was determined by rectal palpation [22] by an experienced veterinarian and animals were recorded as either pregnant or not. Body condition was assessed by palpation of four regions (ribs, hips, spine, and base of tail) each of which are scored on a scale of 1–5 and then averaged across the four regions. This method has been shown to correlate with kidney fat index and total hematocrit [32].

### Serum biochemistry panel protocol

Frozen serum samples were thawed and stored at 4°C for 1–3 days prior to analysis using an Abaxis Vetscan VS2 (Abaxis Inc., Union City, CA, USA) chemistry analyzer and the large animal profile (Abaxis SKU500-023). This machine is maintained by Veterinary Wildlife Services in KNP and is serviced twice a year [33]. The machine is self-calibrating and is equipped with intelligent quality control (iQC) to verify chemistry, optics, and electronic functions of the analyzer during each run. Chemical methods, including sensitivity and specificity for each parameter in the large animal profile, are described by the manufacturer [34]. Parameters measured are described in Table 2. The parameters reported here are stable during transport and storage as we described, with the exception of CK [35]. The half-life of cattle CK is only 4–8 hours [36], but it is known to be stable at -80C [37]. Since our samples were not frozen for several hours after capture, CK values in buffalo serum may be underestimated here.

### Reference intervals

RI were calculated from the managed herd instead of from the free-ranging herd for two reasons: (1) this herd comprised both male and female buffalo across all age groups (calf to geriatric), whereas sampling from the free-ranging herd was age- and sex-targeted; and (2) because the free-ranging herds were not sampled evenly across season. RI were not calculated for the Species360 (captive) buffalo because there were only 20 individuals in the database. RI were calculated using Reference Value Advisor in Microsoft Excel [41] and were calculated following the NCCLS and Clinical and Laboratory Standards Institute (CLSI) guidelines. One randomly selected sample was included in the analysis for animals captured more than one time. Since adults had had significantly different values than calves, RI were calculated using only individuals greater than 5.5 years of age. To restrict our analysis to apparently healthy animals, only individuals with a body condition above 2.5 and that survived for at least 2 more months after capture were included. In conjunction with reference range calculation, we assessed the data for any outliers using the Tukey method [42, 43]. Any identified outliers were removed prior to generating reference rages. The final sample sizes reported exclude these outliers. Due to small sample size, we used a nonparametric robust method on box-cox transformed data to calculate reference intervals [42], similar to the robust approach described by Horn et al [43]. Parametric methods were not used due to small sample size [44]. General linear mixed models (GLMM) demonstrated no significant differences between sexes; therefore, RI were calculated for adults and not separated into male and female intervals. Separate reference intervals were calculated for the wet and dry seasons because of the seasonal variability demonstrated in the GLMM results (Table 3).

**Table 3:** Reference intervals for serum biochemistry parameters of managed adult buffalo.

Parameter	Adult Wet Season (n = 29 animals)	Adult Dry Season (n = 40 animals)
Albumin (g/dL)	3.12–4.54	3.37–4.67
Globulin (g/dL)	3.55–6.25	2.52–5.12
Total protein (g/dL)	7.49–10.15	6.6–9.29
Albumin:Globulin	0.542–1.180	0.761–1.854
Alkaline phosphatase (ALP, U/L)	31.1–240.8	24.3–257.1
Aspartate aminotransferase (AST, U/L)	124.2–279.2	135.6–429.9
γ-Glutamyltransferase (GGT, U/L)	9.2–19.9	6.9–20.6
Creatine kinase (U/L)	0–693.4	233–2926.1
Urea nitrogen (BUN, g/dL)	3.7–15.8	5.9–24.5
Calcium (mg/dL)	7.12–9.41	6.99–9.5
Magnesium (mg/dL)	1.82–4.54	2.07–3.7
Phosphorus (mg/dL)	4.02–9.47	3.09–8.16
Calcium:Phosphorus	0.792–2.066	0.953–2.678

Separate reference intervals were calculated for wet and dry seasons because general linear mixed models showed that biochemistry parameters varied significantly with season.

### Statistical analysis for effects of season, pregnancy, age and sex on biochemistry parameters

GLMM were used to evaluate the effects of age (linear), sex (binomial), season (ordinal), and pregnancy (binomial) on each parameter. Multiple samples per individual were used for this analysis, and animal ID was included as a random effect. Separate models were used for each biochemistry parameter, with the concentration of the parameter as the response variable, age, sex, and season as fixed effects, and animal ID as a random effect. For all models, age was rescaled by centering and dividing by 2 standard deviations to remove the scale effect [45]. Gaussian error distribution was assumed. Akaike’s information criterion (AIC) was used to estimate the relative quality of each model, and then select the model that minimizes loss of information [46]. Marginal R^2^ values were estimated for each of the final models using the MuMIn package [47] following the protocol proposed by Nakagawa et al [48]. Many biochemical parameters vary non-linearly with age such that the parameters tend to be highest or lowest in animals of intermediate age groups [49]. These unimodal associations of focal parameters with age can be approximated by fitting models that include both age and age squared (age^2^) as explanatory variables. As such, we considered effects of both age and age^2^ on our focal parameters, and included age^2^ if it resulted in a model with a significantly better fit based on an ANOVA F-test. Statistical significance was defined as p ≤ 0.05. Statistical analyses were performed in R, using the lmerTest and lme4 packages [50–51].

### Comparison of free-ranging herd and managed herd values

Effects of management (free-ranging herd vs managed herd) were evaluated using separate GLMM that accounted for age and season. Only females greater than 3 years of age were compared between the two herds since the free-ranging herd was an age and gender targeted cohort of buffalo. Age was rescaled for graphing purposes by dividing by two standard deviations to make all the x-axes equivalent. Values for captive (zoo) buffalo from Species360 were obtained, but they are not directly compared due to the small sample size and unknown background of these individuals.

## Results

### Reference intervals

RI for the managed herd were generated for adults during the wet and dry seasons (Table 3). There are large differences in the RI by season, notably AST, GGT, total protein (TP), BUN, CK, and albumin:globulin ratio (Table 3).

### Significance of age, sex, pregnancy status, and season

**Fig 2:**
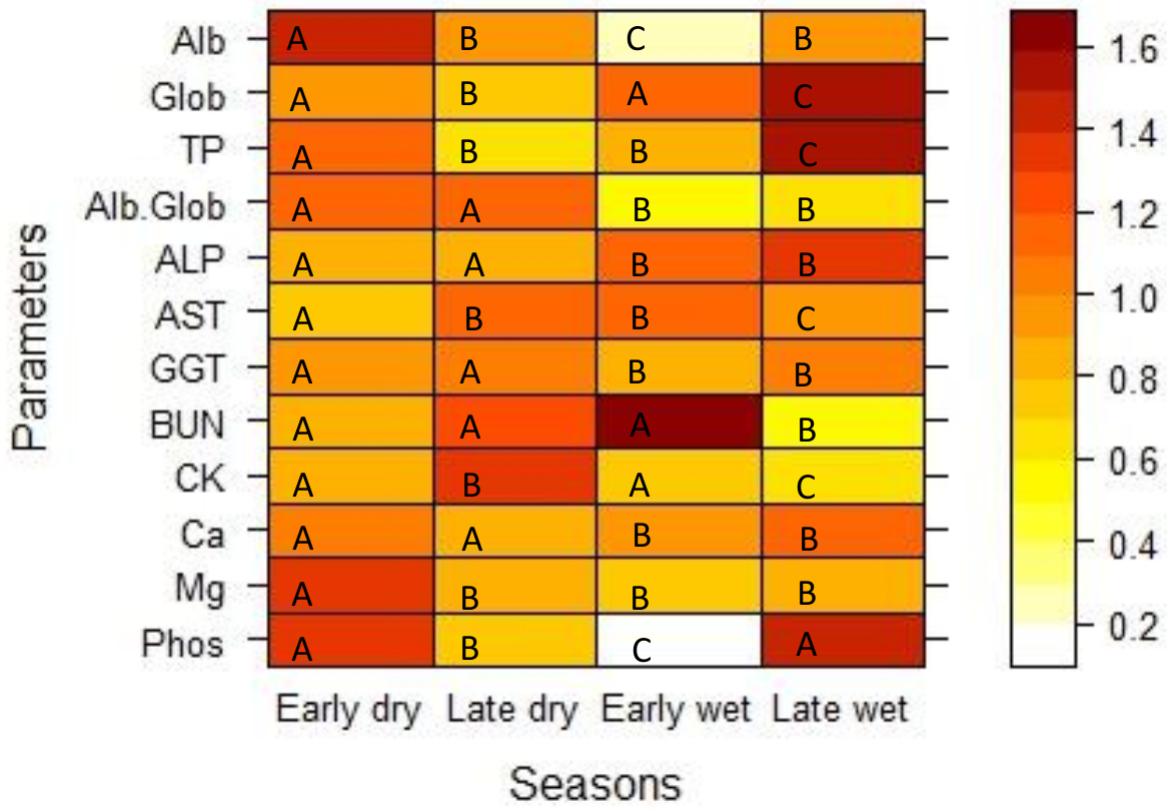
Managed African buffalo serum biochemistry parameters across seasons. Within each parameter, shared letters indicate no significant difference across seasons. The heat map shows the percent difference between seasonal average value and overall average for each parameter, with red being very high concentration and white being very low concentration. All parameters were centered and transformed so that color varies on the same scale for each parameter.

**Fig 3:**
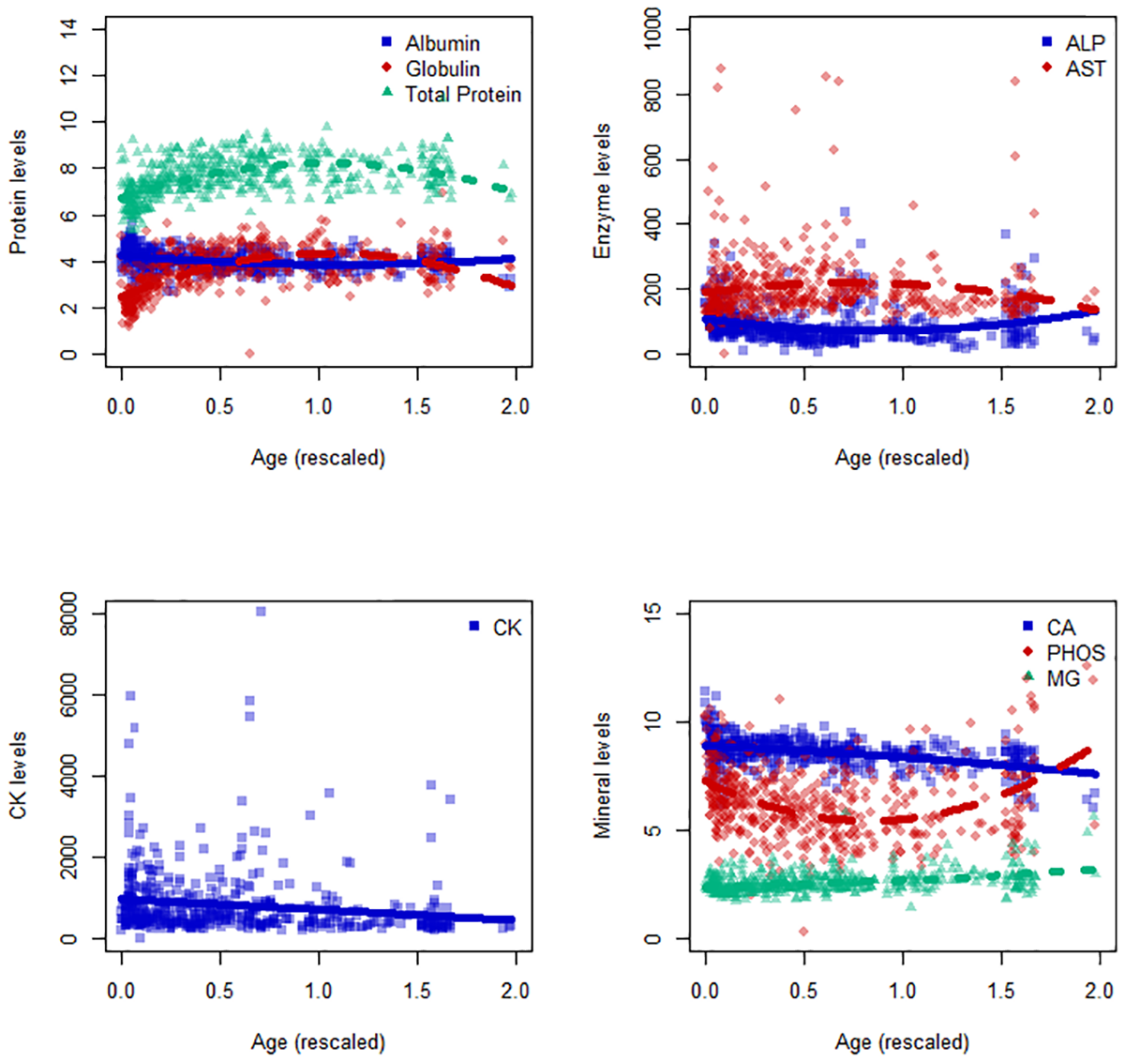
Effects of age on serum biochemistry parameters. Serum biochemistry parameters are only shown here if age or age squared are significant model parameters. The line represents the fitted line from the model while the points are each individual data point. CK is shown on a separate graph due to scale. Age has been rescaled (see Methods).

Sex and pregnancy status did not correlate significantly with any biochemical parameter. For this reason, these variables were not included in the final models. Seasonal variation significantly affected all parameters except GGT (Table 4, Fig 2), although none of the seasonal averages were outside the RI established in this study. Age and/or age^2^ correlated significantly with all biochemical parameters except magnesium, GGT and BUN (Table 4, Fig 3). TP, globulin, ALP, and phosphorus show nonlinear responses with age while albumin, ALP, AST, CK and calcium respond linearly (Table 4, Fig 3). The percent of variance explained by individual differences is provided in Table 4 (marginal R^2^).

**Table 4:** Effects of demographics and season in general linear mixed models for serum biochemistry values in managed buffalo.

Parameter	Age		Age Squared		Season	Marginal R^2^
	Estimate	Significance	Estimate	Significance		
Albumin	-0.77	***	0.37	**	*	0.17
Globulin	3.79	***	-1.79	***	*	0.40
Total protein	3.02	***	-1.43	***	*	0.37
Albumin:Globulin	-2.26	***	1.08	***	*	0.34
Alkaline phosphatase	-1.23	***	0.63	***	*	0.12
Aspartate aminotransferase	0.43	*	-0.28	*	*	0.05
γ-Glutamyltransferase	NS	NS	0.098	0.29	NS	0.17
Creatine Kinase	-0.32	***	NA	NA	*	0.03
Blood Urea Nitrogen	NS	NS	NS	NS	*	0.37
Calcium	-0.38	***	NA	NA	*	0.18
Magnesium	NS	NS	NS	NS	*	0.18
Phosphorous	-4.39	***	2.60	***	*	0.02

General linear mixed models included season and age as fixed effects and animal ID as a random effect. The conditional R2 is an estimate of the percent variation due to random effects, in this case individual differences between animals. For age and age squared, number of asterisks indicates level of significance (* ≤0.05, **≤0.01, ***≤0.001). Season is denoted as significant (*) if there was at least one significant difference between the four seasons. A table (S1 Table) is included in supplementary information that lists the full statistical output for this model.

### Herd comparisons

Although only the managed herd was used to generate reference intervals, data from the free-ranging herd were used to compare the two populations (managed vs. free-ranging) of buffalo. GLMM comparing individual serum biochemical values demonstrated that herd management (managed vs. free-ranging) was a significant variable in all parameters except for ALP and calcium after accounting for age, sex, and season (Table 5). Values for captive animals were obtained from Species360 and are included for reference, but were no direct comparisons were drawn with Species360 values because information on animal age and health status were not reported. Although statistical analysis including Species360 values was not possible, some of the parameters that show increase or decrease between managed and free-ranging buffalo follow the same trend in zoo animals. For instance, albumin decreases from free ranging wild a nimals to the managed herd, and is lower in zoo animals than managed animals. An inverse correlation exists in globulins and BUN, with the highest values in Species360 animals, followed by managed, then by free-ranging animals. Other parameters including calcium, CK, GGT, AST and ALP do not demonstrate a linear relationship from wild to managed to Species360 groups.

**Table 5:** General linear mixed model results comparing wild and managed herds, and mean serum biochemistry values for wild, managed, and captive African buffalo (+/- standard error of the mean). The captive values are from Species360 and are listed in italics as the data was not collected during this project, but is listed for comparison as the only prior information on these values in African buffalo. Asterisks indicate level of significance (* ≤0.05, **≤0.01, ***≤0.001) and parameters with statistically significant differences between managed and wild are bolded.

Parameter	GLM results		Mean values (+/- SEM)		
	Estimate	P-value	Wild	Managed	*Captive* [23]
**Albumin**	0.72	<2e^-16^*	4.6 +/- 0.02	3.89 +/- 0.37	*2*.*1 +/- 0*.*35*
**Globulins**	-1.22	0.00061*	2.29 +/- 0.07	3.57 +/- 0.89	*4*.*9 +/- 0*.*26*
**Total protein**	-1.21	0.03*	6.2 +/- 0.15	7.47 +/- 0.045	*7 +/- 0*.*21553*
Alkaline phosphatase	0.19	0.15	97.44 +/- 2.59	89.2 +/- 3.10	*190 +/- 30*.*67*
**Aspartate aminotransferase**	-0.40	4.03e^-9^***	114.2 +/- 1.62	198.5 +/- 4.92	*187 +/- 21*.*46*
**γ-Glutamyltransferase**	-3.46	1.12e^-5^***	12.21 +/- 0.11	17.43 +/- 0.74	*15 +/- 5*.*20*
**Creatine kinase**	-0.56	1.50e^-8^***	330.1 +/- 7.78	731.5 +/- 46.34	*397 +/- 72*.*95*
**Blood urea nitrogen**	-0.431	<2e^-16^***	8.43 +/- 0.14	13.66 +/- 0.36	*20 +/- 1*.*11*
Calcium	0.38	0.10	8.28 +/- 0.051	8.47 +/- 0.04	*9*.*1 +/- 0*.*19*
**Magnesium**	0.21	0.012*	2.78 +/- 0.02	2.69 +/- 0.08	*2*.*05 +/- 0*.*35*
**Phosphorous**	0.76	0.00015***	6.41 +/- 0.037	5.96 +/- 0.09	*2*.*05 +/- 0*.*44*

## Discussion

In this study, reference intervals were established for 11 serum biochemistry parameters in a herd of African Buffalo kept in a 900ha enclosure within KNP (“managed”). Comparisons were made with a free-ranging population in the same park. Effects of demographic parameters and season on the biochemical parameters were evaluated. Many of the biochemical parameters differed significantly between the managed and free-ranging herds, and also differed from values previously published for captive animals [23]. The provided reference intervals, and the comparative differences observed in this study will be useful for interpreting diagnostic tests.

Season consistently influenced all biochemistry parameters measured, with the exception of GGT. This finding illustrates the central role of environmental factors in mediating the physiology of wild animals. However, variation was not extreme enough to cause means for biochemical parameters in the wet season to fall outside of the RI generated for the dry season, or vice versa. Our results suggest that the observed variation reflects naturally fluctuating physiological shifts that may be due to seasonal variability in nutrition, water availability or disease exposure. Our findings are similar to a study by Yokus et al [12] which found that, in domestic cattle, magnesium and phosphorus levels increased in the spring in response to availability of lush pasture, while total protein levels were higher in the summer due to decreased hydration status.

In African buffalo, there is demonstrated seasonality in disease exposure [52, 53], altered immune defense [54] and altered host behavior due to foraging requirements [55] and reproductive timing [56, 57]. This may explain the seasonal variability in immunoglobulin levels and resultant decreased albumin:globulin ratio. Globulin is strongly associated with immune stimulation and inflammation, while albumin is a negative acute phase protein that decreases in response to inflammation, therefore this ratio is often used as an indicator of recent disease exposure. The decreased albumin:globulin ratio during the wet season could be attributed to either increased disease exposure, or alteration in immune status due to reproductive timing.

Nutritional intake and water availability is greatest at the end of the wet season and the beginning of the dry season, corresponding with increased body condition scores in the buffalo. Body condition scores then decrease throughout the dry season, and may reach conditions near starvation at the end of the dry or early wet season [58]. Seasonal variation was also seen in the physiological parameters reflecting nutritional intake with albumin, phosphorous and magnesium being highest in the wet season. Poor body condition and subclinical dehydration may also affect animals’ response to stress [59]. Dry season conditions result in hypoperfusion of tissues which may predispose them to injury and increase tissue damage during capture events, as reflected in the high levels of CK and AST during the dry season. In addition, decreased hydration during the dry season likely affects renal perfusion causing mild azotemia, as suggested by high blood urea nitrogen concentrations when water was less available.

Somewhat surprisingly, there were no observable effects of sex and reproductive status on any of the biochemistry parameters measured. This latter finding is in contrast to prior studies in other free-ranging ungulates such as fallow deer and collared peccaries, where reproductive status affected ALP and AST, respectively [60, 61]. The lack of a direct effect of sex on biochemical parameters in this study may be due to the sample population, which consisted predominantly of adult females with immature calves and subadults, and only few sexually mature, adult males. Additionally, because females are typically only pregnant once every two years and are lactating in the interim to support calves, there may be too few non-lactating or non-pregnant females for comparison. Moreover, comparing differences between stages of pregnancy (early, mid, late) and/or stages of lactation may lead to altered results. This population did not have enough individuals to assess for this pattern.

In contrast to sex and reproductive status, variation of serum biochemical parameters was consistently demonstrated between different animal ages, although all averages remained within the normal reference intervals. These results demonstrate dynamic shifts in what may be considered normal depending on individual physiology and environment, and are important factors to consider when evaluating an individual biochemical profile. Some of the age-related changes found in this study were predictable based on previous research. Animals are born with underdeveloped immune systems and hepatic function which is reflected by their lower globulins, total protein, and urea nitrogen concentrations. Due to normal bone growth during the first year of life neonates typically have higher phosphorous and ALP concentrations relative to adult animals [62–64]. Several age related changes noted in this study were somewhat surprising, including increased albumin and CK concentrations. Albumin was highest in calves, which may be due to reduced hydration as they compete with larger animals for water access. CK was highest in calves and decreased linearly with age, suggesting that calves experience more acute muscle damage compared to adults during capture events, which may be due to increased trauma during capture and sedation.

Several differences in serum biochemistry values were observed between populations. Specifically, albumin, albumin:globulin ratio, phosphorous, and magnesium were elevated in the wild versus the managed population and globulins were lower in wild animals relative to the managed herd. African buffalo in KNP freely move about in fission fusion societies [65] with dispersal being common [25] and little genetic substructure [66], so it is unlikely that the differences observed between managed and wild herds are due to genetic variation. Additionally, GPS data from the free-ranging herd demonstrates that they ranged broadly throughout the southern portion of the park, and their ranges overlapped with the location of the managed herd [25], so differences in capture location are unlikely to explain differences in serum biochemistry. Biochemical differences may be a result of inadequate nutrition in the managed herd possibly due to reduced ability to migrate to better forage when fenced and debilitation due to increased disease exposure from higher population density [67, 68]. These findings could have implications for management on small game reserves where animals are maintained on limited acreage. AST, GGT, CK, along with urea nitrogen were all significantly elevated in the managed population. Differences in enzyme concentrations may be driven by underlying nutritional differences or other differences in management or capture methods. Values obtained from captive zoo animals [23] differ from the managed and wild herds, and the trends appear consistent with increasing disease and decreasing health in contained populations. However, it is important to remember that the Species360 database contains only opportunistically collected samples, which may be taken when the animal is immobilized to a health problem. Overall, the comparison between the free-ranging, managed, and captive values demonstrates that significant differences in biochemical parameters exist between buffalo populations depending on their management level, and highlights the importance of having reliable RI when assessing the health of an individual or herd.

Results from this study provide RI for a number of important biochemical parameters in African buffalo, and demonstrate the importance of changing environments and management techniques on the physiology of a herd. Moving forward, these RI can be used as a baseline to establish which parameters are the most sensitive and accurate indicators of individual health. This will provide an important tool for evaluating herd health and identifying at risk populations, and adds valuable information to design management strategies to improve overall husbandry.

## Supporting information

S1 Table ANOVA F-test results for each of the final models used to evaluate the effects of season and age on serum concentrations of biochemistry values in managed African buffalo. The F-test compares the model fit with the null model (no predictors other than the intercept) and gives the sum of squared residuals (SSR), numerator degrees of freedom (NumDF), denominator degrees of freedom (DenDF), F values, and probability based on an F distribution (Pr) for each model parameter.
(DOCX)
