# Supplementary material for: Serum biochemistry panels in African buffalo: Defining reference intervals and assessing variability across season, age and sex
Source: PLoS One. 2017 May 4;12(5):e0176830. doi: 10.1371/journal.pone.0176830 (PMC5417560; doi:10.1371/journal.pone.0176830)
Supplement: S1 Table — The F-test compares the model fit with the null model (no predictors other than the intercept) and gives the sum of squared residuals (SSR), numerator degrees of freedom (NumDF), denominator degrees of freedom (DenDF), F values, and probability based on an F distribution (Pr) for each model parameter. (DOCX) [file pone.0176830.s001.docx]

| Biochemistry Value | Age | | | | | Age squared | | | | | Season | | | | | |
| --- | --- | --- | --- | --- | --- | --- | --- | --- | --- | --- | --- | --- | --- | --- | --- | --- |
|  | SSR | NumDF | DenDF | F | Pr | SSR | NumDF | DenDF | F | Pr | SSR | NumDF | DenDF | F | Pr |  |
| Albumin | 1.0430 | 1 | 118.24 | 14.278 | 0.0002486 *** | 0.5777 | 1 | 118.24 | 14.278 | 0.0057929 ** | 8.9085 | 3 | 388.92 | 40.650 | < 2.2e-16 *** |  |
| Globulins | 34.43 | 1 | 117.47 | 125.683 | < 2.2e-16 *** | 18.722 | 1 | 111.53 | 68.342 | 3.206e-13 *** | 39.505 | 3 | 402.19 | 48.071 | < 2.2e-16 *** |  |
| Albumin:Globulins | 8.4368 | 1 | 122.13 | 112.974 | < 2.2e-16 *** | 4.7165 | 1 | 118.39 | 63.158 | 1.248e-12 *** | 4.8707 | 3 | 388.75 | 21.741 | 4.896e-13 *** |  |
| Total protein | 24.839 | 1 | 109.14 | 92.114 | 4.441e-16 *** | 13.547 | 1 | 102.69 | 50.237 | 1.771e-10 *** | 45.119 | 3 | 400.71 | 55.772 | < 2.2e-16 *** |  |
| Alkaline phosphatase | 2.0525 | 1 | 162.26 | 22.957 | 3.716e-06 *** | 1.2835 | 1 | 159.32 | 14.355 | 0.0002142 *** | 4.8838 | 3 | 406.47 | 18.208 | 4.193e-11 *** |  |
| Aspartate aminotransferase | 0.8248 | 1 | 79.81 | 5.3975 | 0.02271 * | 0.868 | 1 | 72.08 | 5.6804 | 0.01979 * | 5.0376 | 3 | 392.86 | 10.9887 | 6.066e-07 *** |  |
| γ-Glutamyltransferase | 0.32004 | 1 | 95.05 | 2.9075 | 0.09143 | 0.12591 | 1 | 85.27 | 1.1438 | 1.1438 | 0.59435 | 3 | 412.68 | 1.7998 | 0.14656 |  |
| Creatine kinase | 4.917 | 1 | 76.06 | 13.760 | 0.0003929 *** | NA | NA | NA | NA | NA | 33.825 | 3 | 399.52 | 31.551 | < 2.2e-16 *** |  |
| Blood urea nitrogen | 0.1253 | 1 | 88.66 | 0.2550 | 0.6148 | 0.0001 | 1 | 76.29 | 0.0001 | 0.9903 | 12.2965 | 3 | 426.49 | 8.3444 | 2.107e-05 *** |  |
| Calcium | 10.2834 | 1 | 85.06 | 42.986 | 4.058e-09 *** | NA | NA | NA | NA | NA | 2.6712 | 3 | 399.34 | 3.722 | 0.01158 * |  |
| Phosphorous | 59.594 | 1 | 115.98 | 45.624 | 6.024e-10 *** | 51.239 | 1 | 108.39 | 39.227 | 7.777e-09 *** | 179.178 | 3 | 410.65 | 45.725 | < 2.2e-16 *** |  |
| Calcium:Phosphorous | 10.741 | 1 | 127.65 | 6.7181 | 0.010656 * | 11.383 | 1 | 111.80 | 7.119 | 0.008757 ** | 16.473 | 3 | 445.13 | 3.4344 | 0.016962 * |  |

**S1 Table: ANOVA F-test results for each of the final models used to evaluate the effects of season and age on serum concentrations of biochemistry values in managed African buffalo.** The F-test compares the model fit with the null model (no predictors other than the intercept) and gives the sum of squared residuals (SSR), numerator degrees of freedom (NumDF), denominator degrees of freedom (DenDF), F values, and probability based on an F distribution (Pr) for each model parameter.
